# *Spirulina maxima* extract prevents activation of the NLRP3 inflammasome by inhibiting ERK signaling

**DOI:** 10.1038/s41598-020-58896-6

**Published:** 2020-02-07

**Authors:** Sungwoo Chei, Hyun-Ji Oh, Ji-Hyeon Song, Young-Jin Seo, Kippeum Lee, Kui-Jin Kim, Boo-Yong Lee

**Affiliations:** Department of Food Science and Biotechnology, College of Life Science, CHA University, Pangyo-ro 335, Bundang-gu, Seongnam-si, Gyeonggi-do 13488 Republic of Korea

**Keywords:** Acute inflammation, Acute inflammation, Inflammasome, Inflammasome, Effectors in plant pathology

## Abstract

The blue-green alga *Spirulina maxima* is a microscopic filamentous cyanobacterium. Spirulina was recently reported to elicit beneficial effects such as reducing cholesterol and inducing weight loss; however, its effects on inflammation are unknown. To determine the effect of *S. maxima* extract (SME) on innate immunity, we investigated the NLRP3 inflammasome activation, which is a multiprotein scaffolding complex that plays important roles in innate immune responses to many pathogenic infections in macrophages. SME suppressed lipopolysaccharide (LPS)-induced upregulation of the pro-inflammatory cytokines tumor necrosis factor-α, interleukin (IL)-12, IL-1β, and IL-18 in RAW264.7 cells. In addition, SME attenuated LPS-induced NLRP3 inflammasome activation, and thus pro-IL-1β could not be cleaved to IL-1β by activated caspase-1, which is activated by the NLRP3 inflammasome in RAW264.7 cells. Moreover, SME inhibited LPS-induced phosphorylation of extracellular signal-regulated kinase (ERK) in RAW264.7 cells, and attenuated the generation of ERK1 induced-reactive oxygen species (ROS), resulting in decreased expression of NF-κB. These findings suggest that SME suppresses the effects of the NLRP3 inflammasome via regulation of extracellular signal-regulated kinase (ERK). In summary, we demonstrated that SME prevents activation of the NLRP3 inflammasome by inhibiting ERK signaling.

## Introduction

Inflammation plays an important role in the innate immune response to pathogen infection^1^. Insufficient or excessive inflammation can lead to various chronic disorders related to the innate immune system. There are distinct innate immune responses to detection of pathogen-associated molecular patterns (PAMPs) and danger-associated molecular patterns^2^. PAMPs activate innate immune responses that protect the host against infection. Bacterial lipopolysaccharides (LPS) are considered to be the prototypical class of PAMP^3^. LPS is a component of the outer cell membrane of Gram-negative bacteria and may activate macrophages^4,5^.

Inflammasome activation is a crucial signaling node mediated by the innate immune system. Inflammasomes are categorized according to their sensor protein: NLRP3 (NOD-like receptor family, pyrin domain-containing 3), AIM2 (absent in melanoma-2), NLRC4 (NOD-like receptor family CARD domain-containing protein 4), and NLRP1 (NOD-like receptor family, pyrin domain-containing 1)^6–8^. The NLRP3 inflammasome is a widely studied multiprotein scaffolding complex^9–11^. LPS stimulation induces activation of the NLRP3 inflammasome^12–14^, which catalyzes the proteolytic maturation of pro-caspase-1 to activated caspase-1^10,15,16^. Unlike other inflammatory cytokines, IL-1β is synthesized in as inactive immature form (pro-IL-1β that must undergo cleavage by caspase-1 to obtain the activated form (IL-1β)^17^.

Macrophages stimulated with LPS activate members of the mitogen-activated protein kinase (MAPKs) family, including extracellular-signal-regulated kinase (ERK), p38, and c-Jun N-terminal kinase (JNK), via several intracellular signaling pathways. LPS stimulation and activation of ERKS, a MAPK protein, result in the generation of nitric oxide (NO) and reactive oxygen species (ROS)^18,19^, which activate NF-κB to regulate NLRP3 inflammasome activation^2,20,21^. ROS generation is attenuated by superoxide dismutase 1 (SOD1) and Glutathione peroxidase 1 (GPx1), both of which are considered antioxidant enzymes.

*Spirulina maxima*, a blue-green alga, is a microscopic filamentous cyanobacterium^22^. Interest in the nutritional value of blue-green algae and their application as a functional food is increasing^23^. *S. maxima* contains high levels of protein, lipids, essential amino acids, minerals, vitamins, photosynthetic pigments, and biologically active substances including phycocyanin, chlorophyll, and β-carotene, and was recently reported to elicit beneficial effects such as inducing weight loss and preventing neurotoxicity^24,25^. However, whether *S. maxima* can reduce inflammation has not been reported. In this study, we used an extract of *S*. maxima (SME) cultured in volcano sea water to determine whether it protects against inflammation. Our results show that *S. maxima* extract (SME) prevents activation of the NLRP3 inflammasome by inhibiting ERK signaling.

## Methods

### Materials and chemicals

Mouse RAW264.7 macrophages and human THP-1 cells were obtained from the American Type Culture Collection (Manassas, VA, USA). Antibodies against inducible nitric oxide synthase (iNOS, #2982), cyclooxygenase-2 (COX-2 #4842), IL-1β (#12426), NLRP3 (#15101), caspase-1 (#3866), p-NF-κB (#3033), NF-κB (#8242), p-IκBα (#2859), IκBα (#4812), p-ERK (#4377), ERK1/2 (#9102), and SOD1 (#2770) were purchased from Cell Signaling (Beverly, MA, USA). Antibodies against caspase-1 (sc-398715), IL-18 (sc-7954), and GPx1/2 (sc-30147) were purchased from Santa Cruz (Santa Cruz, CA, USA).

### Ethics statement

All animals were treated humanely in according to the criteria outlined in the “Guide for the Care and Use of Laboratory Animals” prepared by the National Academy of Science and published by the National Institutes of Health. The experiment was approved by the Institutional Animal Care and Use Committee (IACUC) of CHA University (Seongnam, Kyunggi, Korea: Approval Number 170094).

### Animals and BMDM

Five-week old C57BL/6 mice were purchase from Orient Bio Co. (Gaphyeong, Kyunggi, Korea). Mice were kept under controlled temperature and humidity conditions with a 12 h light/dark cycle. Bone marrow progenitors were harvested from C57BL/6 mice and cultured for 12 d on petri dishes containing RPMI-1640 media supplemented with macrophage colony-stimulating factor, conditioned L929 media. Cells were fed on day 7 and re-plated on tissue culture-treated dishes the day before stimulation to obtain bone marrow derived macrophages (BMDM).

### Preparation of *S. maxima* extract

Samples of *S. maxima* cultivated with volcano sea water in Jeju (Korea) and dried by freeze drying were supplied by the Jeju Research Institute, Korea Institute of Ocean Science & Technology (KIOST). Dried *S. maxima* were subjected to biochemical analysis and microbiological safety standards. The crude biochemical composition of dried *S. maxima* was determined according to the AOAC method^26^, while pigments were analyzed using general methodologies^27^. The biochemical composition of *S. maxima* is shown in Supplementary 1. *S. maxima* extract (SME) was produced by a two-phase method of extraction. In the first step, *S. maxima* extract was ultrasound-extracted in 70% ethanol at room temperature for ~8 h. The product was then ultrasound-extracted at 65–70 °C for ~4 h and the extract was analyzed^22,24^.

### Cell culture and treatment

Cells were maintained in Dulbecco’s modified Eagle’s medium (DMEM) at 37 °C in the presence of 5% CO_2_. The medium contains with 10% fetal bovine serum (FBS) and 1% penicillin/streptomycin. SME was dissolved in DMSO (Sigma-Aldrich, St. Louis, MO, USA). DMSO alone was used as a vehicle control, and cells were washed with PBS and cultured in serum-free medium with SME.

### Immunocytochemistry(ICC) staining

Cells were grown on coverslips (22 × 22 mm) treated with 50 μM SME for 12 h. the cell washed with PBS and fixed with 70% of methanol for 10 min. The coverslips with treated cells are added with Hoechst solution (Sigma-Aldrich) and each antibody. After remove the staining solution and wash the cells three times in PBS. It is examined using a Leica TCS SP5 confocal microscope (GmbH, Wetzlar, Germany).

### Western blotting

Cells were washed with PBS and lysed in cold RIPA buffer supplemented with protease inhibitors (1 mM PMSF, 5 µg/mL aprotinin, and 5 µg/mL leupeptin) and phosphatase inhibitors (1 mM Na_3_VO_4_ and 1 mM NaF). Lysate cells are centrifuged at 12,000 rpm for 5 min at 4 °C and remove the debris. To determine the protein concentration, we used the BCA protein assay (Pierce, Rockford, IL, USA). Bovine serum albumin is used to determine the standard curve. 20 µg of each protein were separated by SDS-PAGE and it is transferred to nitrocellulose membranes (Osmonics, Minnetonka, MN, USA). After transferred the membranes, it was quick washed with Tris-buffered saline containing 0.05% Tween-20 (TBS-T). The membrane incubated overnight at 4 °C with a specific primary antibody 1:1000 diluted in Tris-buffered saline containing 0.05% Tween-20 (TBS-T). The membranes were incubated for 1 h with 5% skim milk and the peroxidase-conjugated IgG added to 5% skim milk for 30 min more. Then, the membranes were washed three times with TBS-T and it is visualized using enhanced chemiluminescence (Amersham Biosciences, Piscataway, NJ, USA) and quantified using the LAS 4000 (GE Healthcare life science, Marlborough, MA, USA). Band densities were determined using ImageJ software (Bethesda, MD, USA).

### RNA isolation and reverse transcription polymerase chain reaction (RT-PCR)

RNA was first extracted by using Trizol reagent (Invitrogen, Carlsbad, CA, USA) and 1 µg of total RNA was reverse transcribed to cDNA using the Maxime RT PreMix kit (Intron, Seongnam, Korea). The sequence of the oligonucleotide primer was as follows; iNOS Forward (5′-TCTCTCGGCCACCTTTGATGAG-3;) and reverse (5′-GGTTGCATCCAGCTTGACCAG-3′); COX-2 Forward (5′-ATGCTCCTGCTTGAG TATGT-3;) and reverse (5′-CACTACATCCTGACCCACTT-3′); IL-12 Forward (5′-CAGAAGCTAACCATCTCCTGGTTTG-3;) and reverse (5′-TCCGGAGTAATTTGGTG CTTCACAC-3′); IL-18 Forward (5′-ATCGCTTCCTCTCGCAACAA-3;) and reverse (5′-CTTCTACTGGTTCAGCAGCCATCT-3′); TNFα Forward (5′-AAGTTCCCAAATGGCCTCCCTCTCATC-3;) and reverse (5′-GGAGGCTGACTTTCTCCTGGTATGAAA-3′); IL-1β Forward (5′-CAGGATGAGGACATGAGCACC-3;) and reverse (5′-CTCTGCACACTCAAACTCCAC-3′); GAPDH Forward (5′-CAGAACTACATCCCTGCATC-3;) and reverse (5′-CCACCTTCCTGATGTCATCA-3′); PCR products were run on 1.5% agarose gels, stained with ethidium bromide and photographed.

### Cellular reactive oxygen species (ROS) assay

ROS were detected using 2′,7′-dichlorofluorescin diacetate (DCFDA; Cellular ROS Detection Assay Kit is used by Abcam (#ab113851, Abcam, Cambridge, UK). According to the manufacturer’s instructions, cells were seeded at 1 × 10^6^ cells/well and collected by trypsinization. Then, the cells were washed twice with PBS, and centrifuged at 1000 rpm for 5 min. Washed cells suspended in 500 µL of PBS. The cells were incubated with 20 µM DCFDA for 30 min at 37 °C. ROS distribution was determined by flow cytometry (FACS Calibur; BD Biosciences, Franklin Lakes, NJ, USA). In another ROS assay using the same assay kit from Abcam, cells were grown on coverslips (22 × 22 mm), treated with 50 μM SME for 12 h. The cells washed with PBS and fixed with 70% of methanol for 10 min. Cells were stained with 20 µM DCFDA for 30 min at 37 °C and examined using a Zeiss LSM880 confocal microscope (Oberkochen, Germany). The data were analyzed with FlowJo (Ashland, OR, USA).

### Transient transfection and luciferase assay

Transient transfection was used with PolyJet (SignaGen, Rockville, MD, USA). According to the manufacturer’s instructions, RAW264.7 cells were cultured 2 × 10^5^ cells/well into the 24well plates. The cells were transfected with 0.5 μg of a plasmid harboring the NF-κB promoter liked to luciferase for 5 h. Another cell transfected with 0.05 μg of the pRL-null vector for 5 h and fresh medium were changed and incubated for 24 h. Thereafter, cells were cultured in serum-free medium containing SME (0, 12.5, 25, and 50 μM) for another 24 h. The cells were harvested with luciferase lysis buffer. Luciferase activity was determined by a dual luciferase assay kit (Promega, Madison, WI, USA).

### Statistical analysis

Data are presented as the mean ± standard deviation (SD) of triplicates. Statistical analyses were performed using a one-way analysis of variance (ANOVA) followed by Tukey’s multiple range test or Mann-Whitney test (SPSS, IBM Armonk, New York, USA). p < 0.05 was considered statistically significant.

## Results

### SME suppresses LPS-induced upregulation of pro-inflammatory cytokines and maturation of pro-IL-1β in RAW264.7 cells

**Figure 1:**
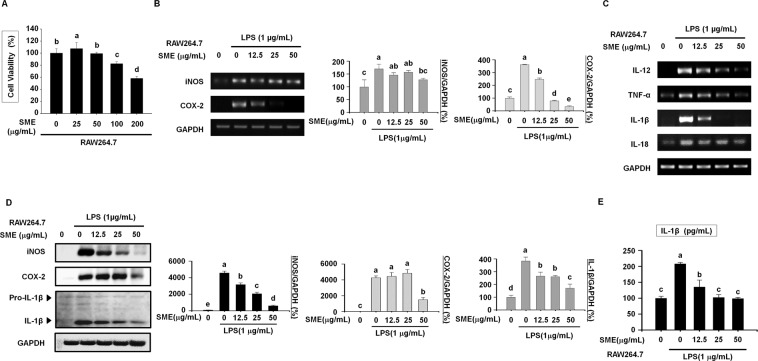
SME suppresses LPS-induced upregulation of pro-inflammatory cytokines and maturation of pro IL-1β in RAW264.7 cells. (**A**) The viability of RAW264.7 cells treated with SME (0, 25, 50, 100, and 200 µg/mL) for 18 h was analyzed by the MTT assay. (**B**) mRNA expression of IL-12 and TNF-α in RAW264.7 cells pretreated with SME (0, 12.5, 25, and 50 µg/mL) for 3 h and then stimulated with LPS (1 µg/mL) for 24 h was assessed by RT-PCR. GAPDH served as a loading control. The graphs show the relative band intensities. Data were quantitatively analyzed using ImageJ software. (**C**) mRNA expression of inflammatory mediators (iNOS and COX-2) and pro-inflammatory cytokines (IL-1β and IL-18) in RAW264.7 cells pretreated with SME (0, 12.5, 25, and 50 µg/mL) for 3 h and then stimulated with LPS (1 µg/mL) for 24 h was assessed by RT-PCR. GAPDH served as a loading control. (**D**) Protein expression of iNOS, COX-2, pro IL-1β and IL-1β in RAW264.7 cells pretreated with SME (0, 12.5, 25, and 50 µg/mL) for 3 h and then stimulated with LPS (1 µg/mL) for 24 h was assessed by western blotting. The graphs show the band intensities. Western blot data were quantitatively analyzed using ImageJ software. (**E**) Secretion of IL-1β by RAW264.7 cells pretreated with SME (0, 12.5, 25, and 50 µg/mL) for 3 h and then stimulated with LPS (1 µg/mL) for 24 h was measured using an ELISA. All data are representative of at least three independent experiments. Statistical significance was determined using a one-way ANOVA followed by Tukey’s post-hoc test. Datasets with the same letter do not significantly differ (p > 0.05, a > b).

We evaluated the cytotoxic effects of SME treatment for 24 h on RAW264.7 mouse macrophages using the MTT assay. SME significantly decreased the viability of RAW264.7 cells in a dose-dependent manner (Fig. 1A). Stimulation with LPS reportedly activates inflammatory mediators such as iNOS and COX-2 in macrophages. mRNA expression of iNOS and COX-2 was determined by reverse transcription PCR (RT-PCR) in RAW264.7 cells pretreated with SME (0, 12.5, 25, and 50 μg/mL) for 3 h and then stimulated with LPS for 24 h. SME dose-dependently suppressed LPS-induced upregulation of iNOS and COX-2 in RAW264.7 cells (Fig. 1B). Next, we investigated the effects of SME on expression of pro-inflammatory cytokines in RAW264.7 cells. RT-PCR demonstrated that SME inhibited LPS-induced upregulation of the pro-inflammatory cytokines IL-12, tumor necrosis factor (TNF)-α, IL-1β, and IL-18 in RAW264.7 cells (Fig. 1C). In addition, western blotting revealed that SME attenuated LPS-induced upregulation of iNOS, COX-2 protein expression in RAW264.7 cells (Fig. 1D). Although the level of pro-IL-1β remained constant irrespective of the SME concentration, increasing the SME concentration attenuated the LPS-induced increase in the level of IL-1β, which is cleaved from pro-IL-1β by caspase-1 (Fig. 1D). Furthermore, an enzyme-linked immunosorbent assay (ELISA) demonstrated that SME dose-dependently inhibited LPS-induced secretion of IL-1β by RAW264.7 cells (Fig. 1E).

### SME inhibits NLRP3 inflammasome-mediated secretion of IL-1β and IL-18

**Figure 2:**
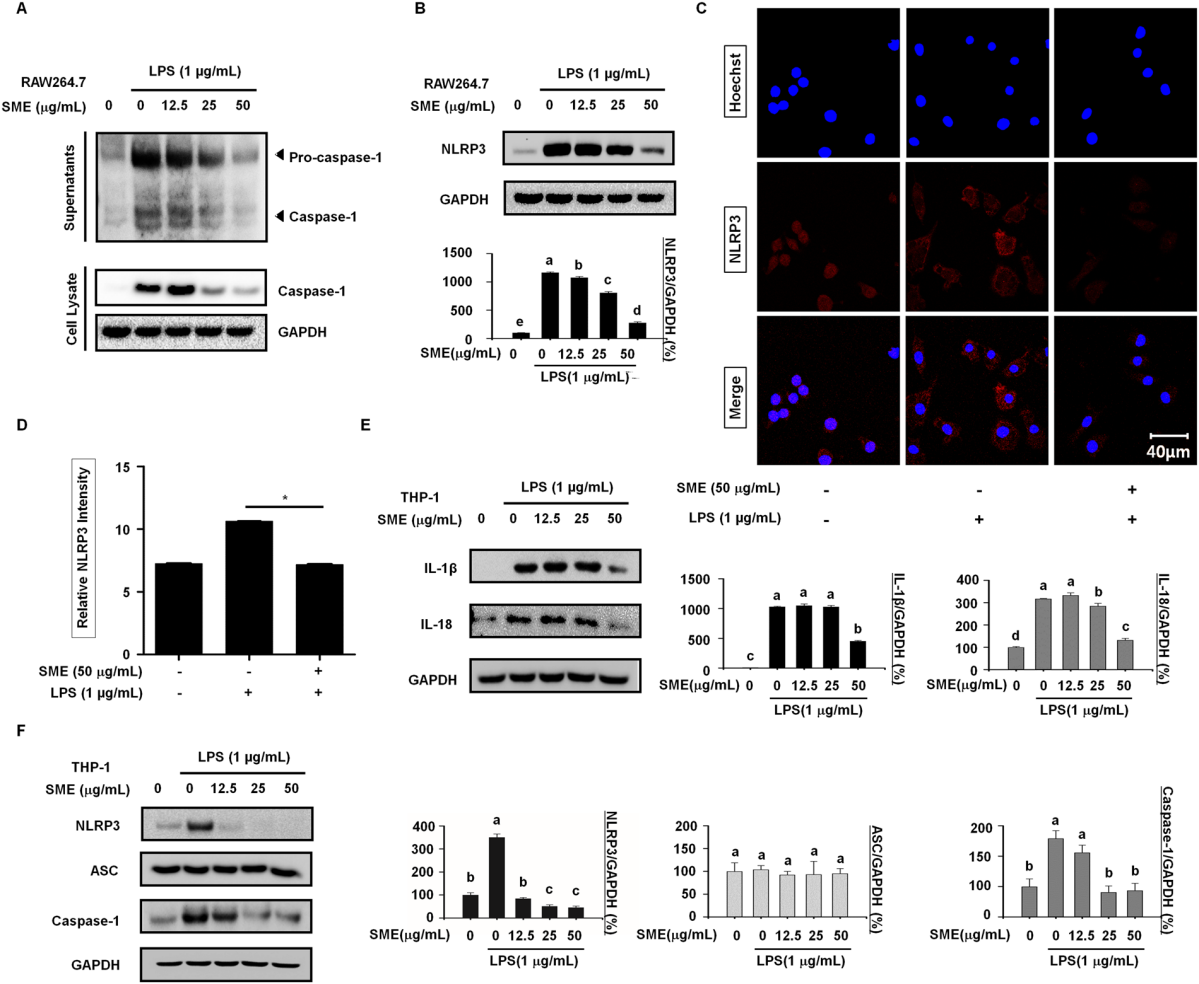
SME inhibits NLRP3 inflammasome-mediated secretion of IL-1β and IL-18.(**A**) RAW264.7 cells were pretreated with SME (0, 12.5, 25, and 50 µg/mL) for 3 h and then stimulated with LPS (1 µg/mL) for 24 h. Supernatants and total cell lysates were analyzed by western blotting. GAPDH served as a loading control. (**B**) Protein expression of NLRP3 in RAW264.7 cells pretreated with SME (0, 12.5, 25, and 50 µg/mL) for 3 h and then stimulated with LPS (1 µg/mL) for 24 h was determined by western blotting. The graph shows the band intensities after normalization of the GAPDH level. (**C**) The localization of NLRP3 in RAW264.7 cells pretreated with SME (50 µg/mL) for 3 h and then stimulated with LPS (1 µg/mL) for 24 h was analyzed by confocal microscopy. Untreated RAW264.7 cells served as a negative control, whereas those treated with LPS (1 µg/mL) alone served as a positive control. (**D**) The numbers of NLRP3-positive cells were determined using ImageJ software and are presented in a bar graph. Data are means ± SD (*p < 0.01, Mann-Whitney test; n = 3, duplicate). (**E**) Protein expression of IL-1β and IL-18 in THP-1 cells pretreated with SME (0, 12.5, 25, and 50 µg/mL) for 3 h and then stimulated with LPS (1 µg/mL) for 24 h was determined by western blotting. The graphs show the band intensities relative to that of GAPDH. (**F**) Protein expression of NLRP3, ASC, and caspase-1 in THP-1 cells pretreated with SME (0, 12.5, 25, and 50 µg/mL) for 3 h and then stimulated with LPS (1 µg/mL) for 24 h was determined by western blotting. The graphs show the band intensities after normalization of the GAPDH level. All data are representative of at least three independent experiments. Statistical significance was determined using a one-way ANOVA followed by Tukey’s post-hoc test. Datasets with the same letter do not significantly differ (p > 0.05, a > b).

The NLRP3 inflammasome, which comprises caspase-1, ASC, and NLRP3, is involved in secretion of IL-1β and IL-18. We investigated whether SME suppresses LPS-induced secretion of IL-1β and IL-18 by modulating the NLRP3 inflammasome. To this end, we immunoblotted the conditioned medium and lysate of RAW264.7 cells for pro-caspase-1 and caspase-1. SME suppressed LPS-induced activation of caspase-1 in RAW264.7 cells (Fig. 2A). Moreover, SME dose-dependently decreased LPS-induced upregulation of NLRP3 in RAW264.7 cells (Fig. 2B). This finding was confirmed by confocal microscopy analysis of NLRP3 in RAW264.7 cells (Fig. 2C). The fluorescence intensity of NLRP3 staining was significantly lower in cells pretreated with SME prior to LPS stimulation than in cells treated with LPS alone (Fig. 2D). We used human THP-1 macrophages to confirm that SME suppresses LPS-induced release of IL-1β and IL-18. SME attenuated LPS-induced release of IL-1β and IL-18 in THP-1 cells (Fig. 2E). Moreover, LPS increased expression of NLRP3 and caspase-1, but not of ASC, in THP-1 cells and these increases were inhibited by SME (Fig. 2F). These results suggest that ASC is not required for release of pro-inflammatory cytokines mediated by the NLRP3 inflammasome.

### SME attenuates LPS-induced NF-κB activation, nitric oxide (NO) production, and ROS generation in RAW264.7 cells

**Figure 3:**
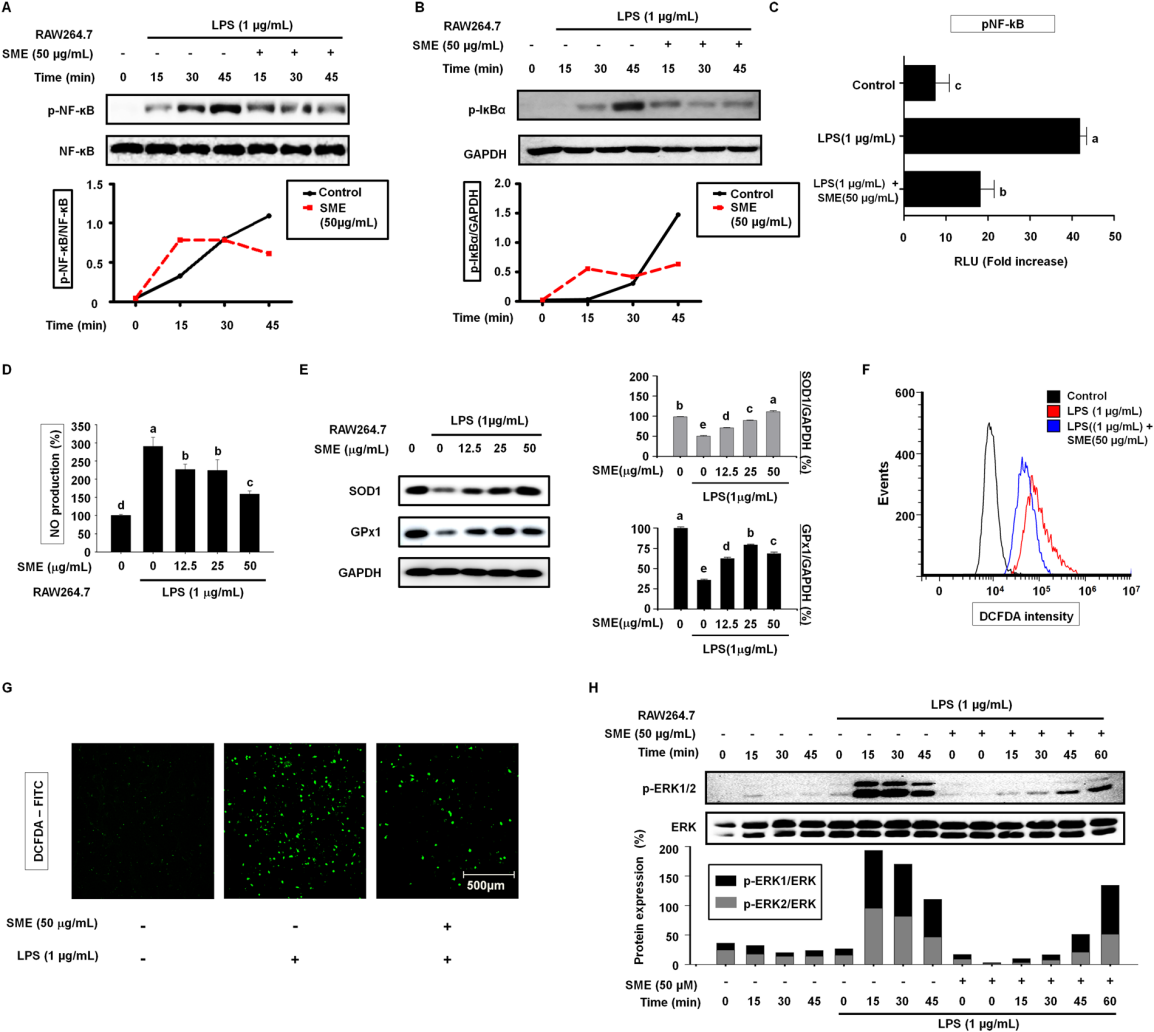
SME inhibits LPS-induced ROS generation and NF-κB signaling. (**A**) Expression of p-NF-κB and NF-κB in RAW264.7 cells pretreated with SME (50 µg/mL) for 3 h and then stimulated with LPS (1 µg/mL) for the indicated duration was determined by western blotting. The NF-κB were used as loading controls for p-NF-ĸB. (**B**) Expression of p-IκBα in RAW264.7 cells pretreated with SME (50 µg/mL) for 3 h and then stimulated with LPS (1 µg/mL) for the indicated duration was determined by western blotting. GAPDH was used as aloading control for p-IĸBα. (**C**) NF-ĸB promoter activity was determined in confluent RAW264.7 cells transiently transfected with the NF-ĸB or empty pGL-basic luciferase reporter construct, pretreated with SME (50 µg/mL) for 3 h, and then stimulated with LPS (1 µg/mL) for 18 h. (**D**) The level of NO in the culture media of RAW264.7 cells pretreated with SME (0, 12.5, 25, and 50 µg/mL) for 3 h and then stimulated with LPS (1 µg/mL) for 24 h was estimated using Griess reagent. (**E**) Expression of GPx1 and SOD1 in RAW264.7 cells pretreated with SME (0, 12.5, 25, and 50 µg/mL) for 3 h and then stimulated with LPS (1 µg/mL) for 18 h was determined by western blotting. GAPDH was used as a control. Data were quantitatively analyzed using ImageJ software. (**F**) The level of ROS in RAW264.7 cells pretreated with SME (50 µg/mL) for 3 h and then stimulated with LPS (1 µg/mL) for 24 h was determined by flow cytometry following incubation with DCFH-DA for 60 min. (**G**) Images of RAW264.7 cells incubated with the ROS-reactive dye DCFH-DA to assess the level of ROS. Green fluorescence indicates the presence of DCF oxidized from DCFH. Higher fluorescence intensity indicates higher ROS levels. ROS production was induced by incubation with LPS (1 µg/mL) for 18 hours in the presence or absence of SME (50 µg/mL). (**H**) The level of ERK phosphorylation in RAW264.7 cells pretreated with SME (50 µg/mL) for 3 h and then stimulated with LPS (1 µg/mL) for the indicated duration was determined by western blotting. The graph shows the p-ERK1/ERK2 ratio after normalization of the total ERK level. All data are representative of at least three independent experiments. Statistical significance was determined using a one-way ANOVA followed by Tukey’s post-hoc test. Bars with the same letter do not significantly differ (p > 0.05, a > b).

Stimulation of macrophages with LPS activates NF-κB, which induces expression of NLRP3. Therefore, we hypothesized that NF-κB is involved in the effects of SME on LPS-stimulated RAW264.7 cells. To investigate this, we monitored activation of NF-κB over time in RAW264.7 cells by western blotting. The intensity of the p-NF-κB band increased up to 45 min in cells treated with LPS alone, but peaked at 15 min and subsequently decreased in cells pretreated with 50 µg/mL SME prior to LPS stimulation (Fig. 3A). The decrease of NF-κB from nucleus protein synthesis was exported out of the nucleus synthesis IκBα. SME attenuated LPS-induced upregulation of p-IκBα with a similar time pattern (Fig. 3B). Transcriptional activity of NF-κB was studied using a reporter gene assay. NF-κB promoter activity was significantly increased in cells treated with LPS alone and this effect was significantly attenuated by pretreatment with SME (Fig. 3C). ROS have been proposed to activate NF-κB and to oxidize its subunits. Moreover, ROS play an important role in signal transduction, gene expression, and cell proliferation, and are involved in regulation of the biologically effective concentration of NO. There is a delicate balance between the levels of NO and ROS in cellular systems. We investigated NO production using Griess reagent. LPS increased NO production and this effect was suppressed by pretreatment with SME (Fig. 3D). When RAW264.7 cells were pretreated without or with increasing concentrations SME for 3 h and then treated with LPS for 18 h, western blot analysis showed that increasing SMA concentrations prevented the LPS-induced decrease in SOD1 and GPx1 expression observed in the absence of SMA (Fig. 3E). To determine whether SME has an antioxidant effect on RAW264.7 cells stimulated with LPS, DCFH-DA, the most widely used probe to detect intracellular H_2_O_2_, was used for flow cytometry (Fig. 3F) and confocal microscopy (Fig. 3G). DCFH-DA fluorescence was increased by LPS stimulation and this effect was attenuated by pretreatment with SME (Fig. 3F,G).

LPS activates ERK1/2, which is a MAPK. Western blotting demonstrated that LPS induced ERK phosphorylation in RAW264.7 cells in a time-dependent manner (Fig. 3F). The level of p-ERK peaked after LPS treatment for 15–30 min and decreased thereafter. SME inhibited LPS-induced phosphorylation of ERK after 15–30 min, and the amount of p-ERK had returned to the basal level after 60 min in LPS-stimulated RAW264.7 cells pretreated with SME

### SME suppresses expression of signaling components involved in LPS-induced activation of ERK in RAW264.7 cells

**Figure 4:**
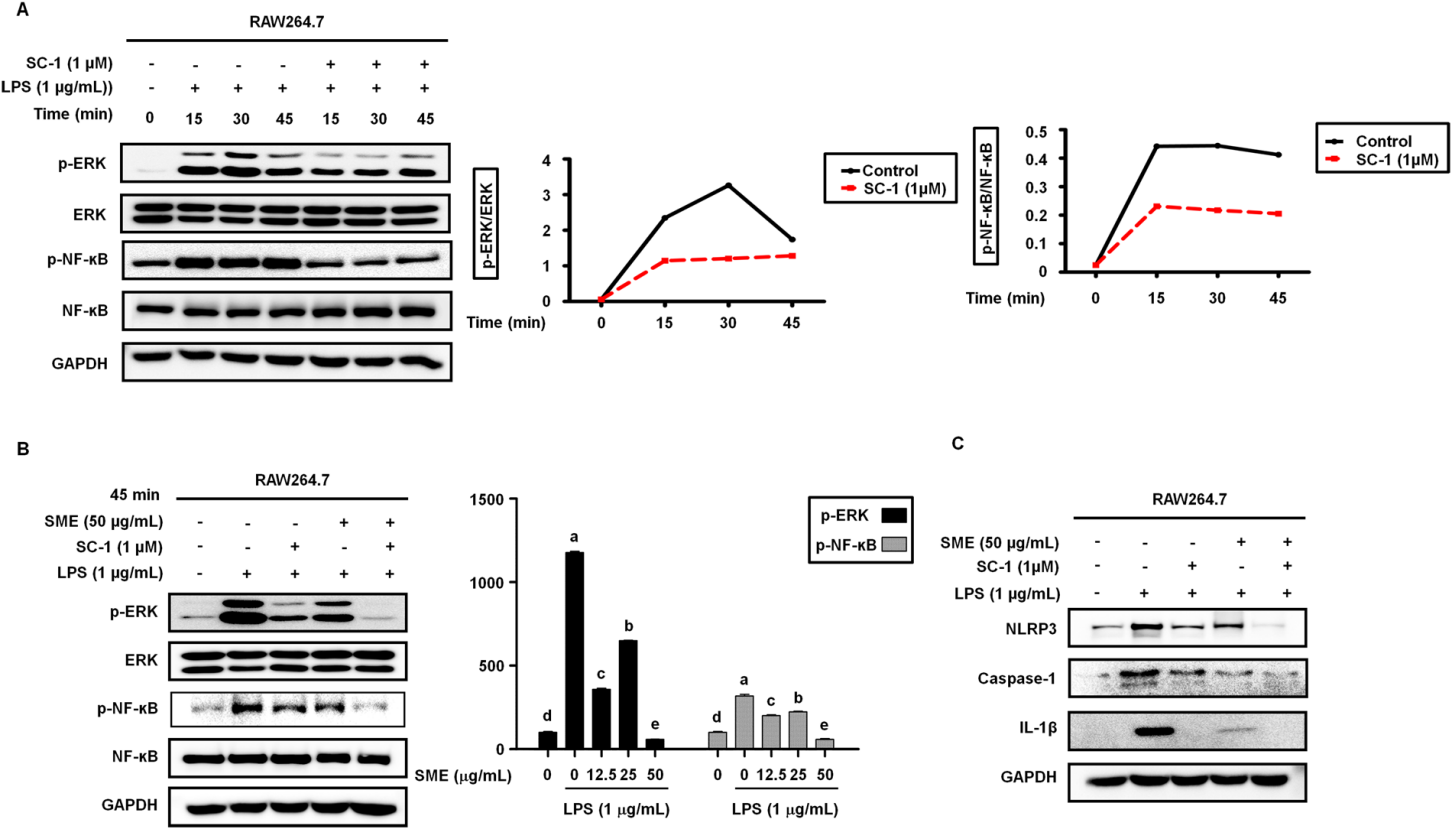
SME suppresses expression of signaling components involved in LPS-induced activation of ERK in RAW264.7 cells. (**A**) Activation of NF-κB and ERK in RAW264.7 cells incubated with the ERK inhibitor SC-1 (1 µM) for 3 h and then simulated with LPS (1 µg/mL) for the indicated durations was determined by western blotting. The graphs show induction of p-NF-κB and p-ERK1/2 expression by LPS over time.(**B**) Phosphorylation of ERK and NF-κB in RAW264.7 cells pretreated with SME (50 µg/mL) in the absence or presence of SC-1 (1 µM) and then stimulated with LPS (1 µg/mL) for 45 min was determined by western blotting. The graph shows the relative intensities of the p-NF-κB and p-ERK bands after normalization of the total NF-κB and ERK levels, respectively. (**C**) Protein expression of NLRP3, caspase-1, and IL-1β in RAW264.7 cells pretreated with SME (50 µg/mL) for 3 h in the absence or presence of SC-1 (1 µM) and then stimulated with LPS (1 µg/mL) for 24 h was determined by western blotting. Data are means ± SD of three independent experiments. Statistical significance was determined using a one-way ANOVA followed by Tukey’s post-hoc test (p > 0.05, a > b).

To confirm that LPS-induced ERK activation was linked to NF-kB signaling, we monitored activation of NF-kB and ERK over time in RAW264.7 cells preincubated with or without 1 µM SC-1, an inhibitor of ERK activation, for 3 h and then treated with 1 μM LPS for 15–45 min using western blotting. Figure 4 shows that LPS-induced phosphorylation of ERK (p-ERK), which peaked after 30 min, was inhibited by SC-1 pretreatment (Fig. 4A). Similarly, the LPS-induced phosphorylation of NF-kB (p-NF-kB), which peaked after 15 min, was also inhibited by SC-1 pretreatment (Fig. 4A). When RAW264.7 cells were pretreated with 50 µg/mL SME in the absence or presence of 1 µM SC-1 and then stimulated with LPS for 45 min, SC-1 or SME inhibited LPS-induced phosphorylation of ERK and NF-kB. However, when RAW264.7 cells were treated with SC-1 and SME together, phosphorylation of ERK and NF-kB was much lower than that seen when RAW264.7 cells were treated with SC-1 or SME (Fig. 4B). Similar results were obtained when the effects of SC-1 and SME either alone or together on NLRP3, caspase-1 and IL-1β activation were examined (Fig. 4C).

### SME suppresses LPS-induced activation of the NLRP3 inflammasome in an ERK-dependent manner in BMDMs

**Figure 5:**
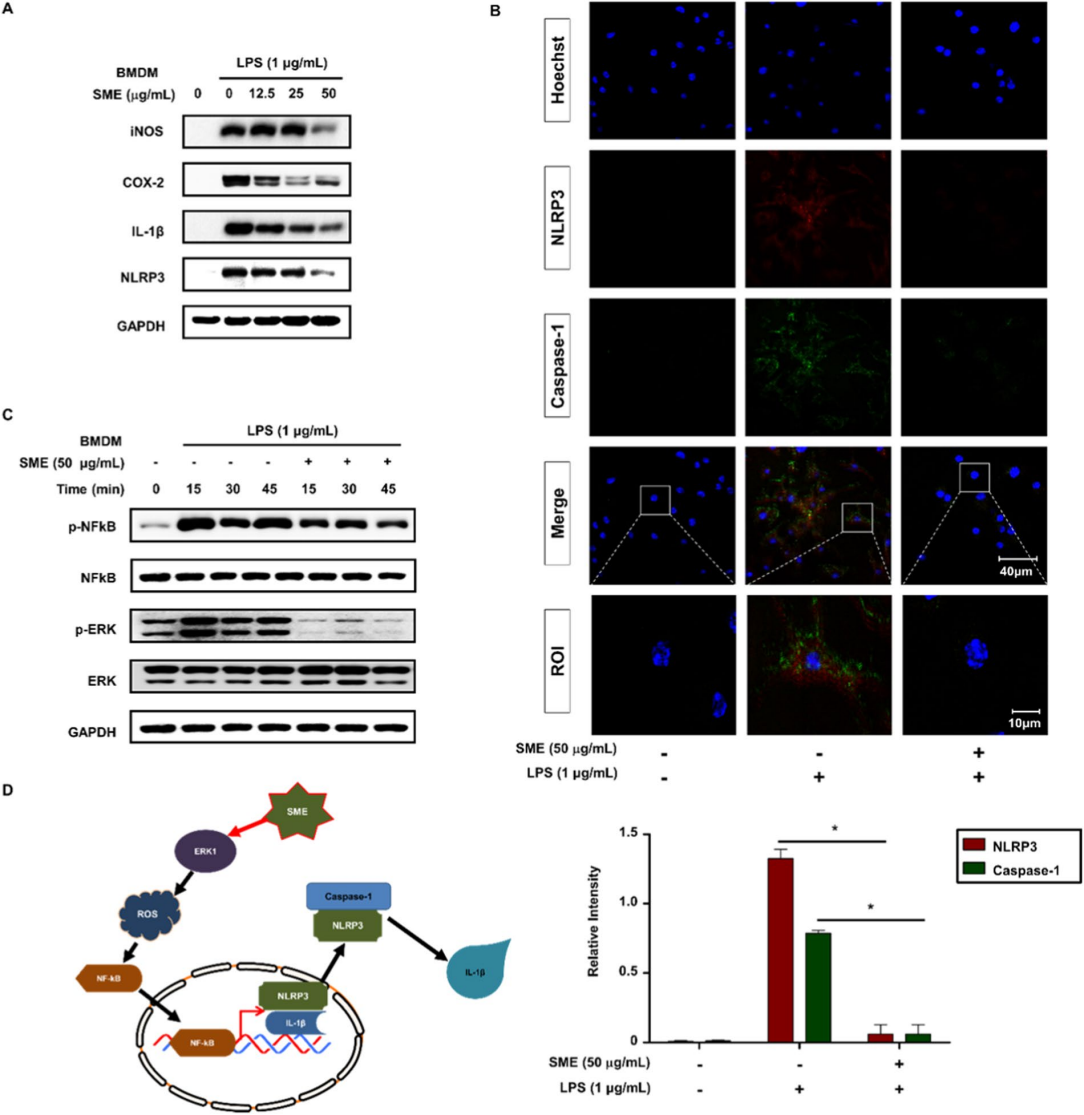
SME suppresses LPS-induced activation of the NLRP3 inflammasome in an ERK-dependent manner in BMDMs. (**A**) Protein expression of iNOS, COX-2, IL-1β, and NLRP3 in BMDMs pretreated with SME (0, 12.5, 25, and 50 µg/mL) for 3 h and then stimulated with LPS (1 µg/mL) for 24 h was determined by western blotting. GAPDH served as a loading control. (**B**) The subcellular localizations of NLRP3 (red) and caspase-1 (green) in BMDMs pretreated with or without SME (50 µg/mL) for 3 h and then stimulated with LPS (1 µg/mL) for 24 h were determined by confocal microscopy. Regions of interest (ROI) are enlarged. Images are representative of three independent experiments. Fluorescence intensities were quantified using ImageJ software. Data are means ± SD (*p < 0.01, Mann-Whitney test; n = 3, duplicate). (**C**) Activation of NF-ĸB and ERK in BMDMs pretreated with SME (50 µg/mL) for 3 h and then stimulated with LPS (1 µg/mL) for the indicated durations was analyzed by western blotting. (**D**) Schematic model.

We next evaluated the effects of SME treatment on Bone Marrow Derived Macrophages (BMDMs). Western blotting revealed that treatment of BMDMs with SME for 24 h attenuated LPS-induced upregulation of iNOS, COX2, IL-1β, and NLRP3 protein expression (Fig. 5A). This finding was confirmed by confocal microscopy analysis of NLRP3 and Caspase-1 in BMDMs. The fluorescence intensities of NLRP3 and Caspase-1 staining were significantly lower in cells pretreated with 50 µg/mL SME prior to LPS stimulation than in cells treated with LPS alone (Fig. 5B). Stimulation of macrophages with LPS activates NF-κB, which induces expression of NLRP3. When NF-κB activation was monitored over time in BMDMs by western blotting, we found that the intensity of the p-NF-κB band, which increased up to 45 min in cells treated with LPS alone, was lower in cells pretreated with 50 µg/mL SME prior to LPS stimulation (Fig. 5C). Western blotting demonstrated that LPS-induced ERK phosphorylation in BMDMs in a time-dependent manner. SME attenuated LPS-stimulated NLRP3 inflammasome activation in an ERK-dependent manner in BMDMs.

## Discussion

Inflammation plays a very important role in the innate immune response to pathogen infection, protects against harmful stimuli, and is involved in the pathogenesis of many diseases^28^. LPS, a prototypical class of PAMPs, is a component of the outer cell membrane of Gram-negative bacteria^29,30^. This study demonstrates that SME prevents LPS-induced activation of the NLRP3 inflammasome and secretion of pro-inflammatory cytokines in macrophages. We measured the cytotoxic effects of SME using the MTT assay to determine the optimal concentration. SME was used at concentrations of up to 50 µg/mL because treatment with concentrations of 100 µg/mL or higher elicited significant cytotoxic effects. To investigate the anti-inflammatory effect of SME, we pretreated RAW264.7 cells with SME for 3 h prior to LPS stimulation. SME attenuated LPS-induced upregulation of inflammatory mediators such as iNOS and COX-2 as well as pro-inflammatory cytokines in a dose-dependent manner. Specifically, SME attenuated LPS-induced upregulation of TNF-α (pro-inflammatory cytokine) and IL-12, which stimulates production of TNF-α. In addition, SME suppressed LPS-induced maturation of pro-inflammatory cytokines, such as the conversion of inactive pro-IL-1β to active IL-1β. These data indicate that SME reduces the secretion of inflammatory cytokines induced by the NLRP3 inflammasome in a dose-dependent manner. The anti-inflammatory effects of SME were analyzed when cells were pretreated with SME for 3 h and then stimulated with LPS for 24 h.

We monitored activation of ERK, NF-κB, and NLRP3 in macrophages, as well as IL-1β secretion. Western blotting and immunocytochemistry showed that SME attenuated LPS-induced activation of caspase-1 and NLRP3. However, because ASC is not expressed in RAW264.7 cells, we could not investigate the role of ASC in SME attenuation of LPS-induced NLRP3 inflammasome activation. This should be investigated in a future study.

To confirm NLRP3 inflammasome activation, we investigated the effects of LPS and SME on THP-1 human macrophages, which express ASC. LPS increased the expression of NLRP3 and caspase-1, but not ASC, and SME attenuated these increases in a dose-dependent manner. This indicates that SME inhibits NLRP3 inflammasome activation regardless of ASC expression.

Inactive NF-κB in the cytosol is in a complex with IκBα. LPS stimulation initiates phosphorylation and activation of I-κB kinase (IKK), which in turn phosphorylates I-κBα, resulting in its dissociation from NF-κB and translocation of NF-κB to the nucleus where it induces NLRP3 expression^31,32^. NF-κB signaling plays an important role in regulating the NLRP3 inflammasome. We analyzed phosphorylation of NF-κB by western blotting for up to 45 min. SME dramatically attenuated LPS-induced phosphorylation of NF-κB at 45 min. SME attenuates LPS-induced activation of the NLRP3 inflammasome by modulating NF-κB signaling. Moreover, SME had similar effects on LPS-induced phosphorylation of IκBα.

ERK1/2, a member of the MAPK family, is an important mediator of the LPS/TLR4 signaling involved in the activation of the NLRP3 inflammasome. LPS-induced phosphorylation of ERK was inhibited by SME after 15–30 min. Since LPS stimulation and phosphorylation of ERK1 induce ROS generation, we investigated the antioxidant effect of SME by measuring the levels of SOD1, which attenuates the harmful effects of ROS, and GPx1, which removes toxic hydrogen peroxide after treatment with SME. The results showed that SME attenuated the LPS-induced decrease in SOD1 and GPx1 expression in RAW264.7 cells.

Phosphorylation of ERK peaked at 60 min after LPS treatment. Therefore, western blotting samples were collected at 0–60 min. Treatment with the ERK1 inhibitor SC-1 significantly blocked activation of the NLRP3 inflammasome, confirming that ERK1 is a key protein in the activation of the NLRP3 inflammasome.

In conclusion, this study demonstrates that SME protects against LPS-induced inflammation in mouse macrophages, human macrophages, and bone marrow-derived macrophages (BMDMs). SME attenuated LPS-induced activation of the NLRP3 inflammasome in an ERK-dependent manner. Further elucidation of the mechanisms involved may assist in the treatment of inflammasome-dependent disorders.

## Supplementary information

Supplementary information
