# Supplementary material for: Spirulina maxima extract prevents activation of the NLRP3 inflammasome by inhibiting ERK signaling
Source: Sci Rep. 2020 Feb 7;10:2075. doi: 10.1038/s41598-020-58896-6 (PMC7005707; doi:10.1038/s41598-020-58896-6)
Supplement: Supplementary file 1 — Supplementary information [file 41598_2020_58896_MOESM1_ESM.pdf]

Supplementary information to *Spirulina maxima* extract prevents activation of the NLRP3 inflammasome by inhibiting ERK signaling

Sungwoo Chei[1], Hyun-Ji Oh[1], Ji-Hyeon Song[1], Young-Jin Seo[1], Kippeum Lee[1], Kui-Jin Kim[1], and Boo-Yong Lee[1*]

[1]Department of Food Science and Biotechnology, College of Life Science, CHA University, Pangyo-ro 335, Bundang-gu, Seongnam-si, Gyeonggi-do, Republic of Korea, 13488
[*]Correspondence and requests for materials should be addressed to B.Y.L. (email: bylee@cha.ac.kr)

**Component**	
**Protein (%)**	**55.3**
**Carbohydrate (%)**	**30.5**
**Lipid (%)**	**0.9**
**Ash (%)**	**11.3**
**Chlorophyll-α (mg/g)**	**69.9**
**Phycocyanin (mg/g)**	**6.5**

Supplementary 1. Biochemical composition of *S. maxima*

The protein, carbohydrate, lipid, and ash contents of *S.maxima* based as percentages of dried weight were 55.3%, 30.5%, 0.9%, and 11.3%, respectively. The average concentrations of phycocyanin and chlorophyll-α were 69.9 and 6.5 mg/g, respectively. *Escherichia coli* was not detected. Levels of all heavy metals (Pb, Cd, Hg, and As) were within the limits specified by the guidelines of the Korean Ministry of Food and Drug Safety.
